# Oral Nitrate-Reducing Bacteria and Nitric Oxide Homeostasis: Functional Host–Microbe Interactions in Oral and Vascular Health

**DOI:** 10.3390/ijms27209012

**Published:** 2026-10-11

**Authors:** Serena Altamura, Francesca Lombardi, Alessia Ciafarone, Paola Palumbo, Benedetta Cinque, Maria Grazia Cifone, Davide Pietropaoli

**Affiliations:** 1 Department of Physical and Chemical Sciences, University of L’Aquila, 67100 L’Aquila, Italy; alessia.ciafarone@univaq.it; 2 Department of Life, Health & Environmental Sciences, University of L’Aquila, 67100 L’Aquila, Italypaola.palumbo@univaq.it (P.P.); benedetta.cinque@univaq.it (B.C.); mariagrazia.cifone@univaq.it (M.G.C.); davide.pietropaoli@univaq.it (D.P.)

**Keywords:** oral microbiome, oral nitrate-reducing bacteria, nitric oxide homeostasis, nitrate–nitrite–NO pathway, host–microbe interactions, nitric oxide bioavailability, oral dysbiosis, periodontal disease, vascular health, blood pressure

## Abstract

Nitric oxide (NO) is a key regulator of vascular homeostasis and host–microbe interactions. In addition to the endogenous nitric oxide synthase (NOS) pathway, the enterosalivary nitrate–nitrite–NO pathway provides an alternative source of NO and critically depends on oral nitrate-reducing bacteria (ONRB). This review examines the oral microbiome–NO axis and its role in nitrate metabolism, NO homeostasis, and oral–systemic interactions. A narrative review integrating microbiological, mechanistic, and clinical evidence on oral nitrate metabolism, ONRB activity, and their impact on oral and systemic outcomes, including dietary and antiseptic interventions. The functional activity and nitrate-reducing capacity of the oral microbiome are key determinants of nitrate reduction and systemic NO bioavailability. Oral dysbiosis, periodontal disease, and antiseptic mouthwash use impair ONRB activity and nitrite production, potentially affecting vascular function and blood pressure. Conversely, dietary nitrate enhances ONRB activity and nitrate-to-nitrite conversion, supporting oral and vascular health. Interindividual variability, partly influenced by sex-related factors, may contribute to heterogeneous responses. The oral microbiome–NO axis represents a functional interface between host physiology and microbial metabolism, linking oral ecology with vascular regulation. Through their activity, ONRB contribute to NO homeostasis, highlighting nitrate bioactivation as an important component of host–microbe interactions with implications for oral and systemic physiology. Recognition of this pathway expands current understanding of host–microbe cooperation in NO homeostasis and may inform future microbiome-based approaches to support oral and cardiovascular health.

## 1. Introduction

Nitric oxide (NO) plays a central role in vascular homeostasis by supporting endothelial function, vasodilation, and cardiometabolic balance [1]. Under physiological conditions, NO is primarily generated through the oxygen-dependent L-arginine/NO synthase (NOS) pathway. However, in common clinical settings such as hypertension, aging, chronic inflammation, and oxidative stress, NOS-dependent NO synthesis may become impaired [2]. In these contexts, the enterosalivary nitrate–nitrite–NO pathway represents an important complementary source of NO bioavailability. This pathway critically depends on oral nitrate-reducing bacteria (ONRB), which mediate the microbial reduction in nitrate to nitrite, a key step in nitrate bioactivation [3]. By enabling nitrite formation through specialized nitrate-reducing systems, ONRB function as an integral extension of host NO metabolism, linking oral microbial activity with systemic NO homeostasis [3]. Disruption of oral microbial ecology, as observed in oral dysbiosis, promotes a shift toward anaerobic and pro-inflammatory communities and reduces both the abundance and functional activity of ONRB [4].

Interventional studies provide compelling evidence linking oral microbial function, nitrate metabolism, and cardiovascular physiology. Suppression of oral bacteria through antiseptic mouthwashes, particularly chlorhexidine-based formulations, reduces salivary and plasma nitrite levels and has been shown to acutely increase blood pressure (BP) [5,6]. In contrast, nitrate-rich diets enhance nitrate-to-nitrite conversion, improve endothelial responsiveness, and lower BP, particularly in nitrate-responsive individuals [7,8,9,10,11,12,13]. More recently, emerging evidence suggests that nitrate-reducing probiotics may further enhance nitrate bioactivation, highlighting their potential as microbiome-targeted modulators of this pathway [14,15].

Despite growing recognition of this pathway, its integration into current models of oral and systemic health remains incomplete. In particular, the contribution of microbial nitrate metabolism to periodontal health, systemic inflammation, and cardiometabolic risk remains unclear, especially given substantial interindividual variability driven by microbial, behavioral, and host-related factors [16,17,18]. Moreover, biological sex, well recognized as a modifier of vascular physiology, immune regulation, and microbiome composition, remains insufficiently explored in the context of oral nitrate metabolism despite emerging evidence suggesting sex-related differences in nitrate reduction and NO bioavailability [19,20].

In this review, we examine the oral microbiome–NO axis as a functional link between oral ecology and systemic physiology. Throughout the review, oral health is considered in terms of the composition and functional activity of the oral microbiome, periodontal health, and local inflammatory status, whereas vascular health encompasses endothelial function, BP regulation, vascular homeostasis, and cardiovascular risk markers associated with NO bioavailability. We integrate ecological, microbiological, mechanistic, and clinical evidence, highlight converging findings from dietary and antiseptic intervention studies, and discuss emerging responder phenotypes and sex-related differences. Finally, we identify current knowledge gaps and outline microbiome-targeted strategies with potential relevance for oral and cardiovascular health. Beyond descriptive associations, we propose that impaired microbial nitrate reduction may represent a distinct mechanistic axis contributing to the oral–systemic link, alongside inflammation and microbial dissemination. Unlike previous reviews that broadly discuss the relationship between the oral microbiome and systemic health [21], the present review specifically focuses on ONRB and the enterosalivary nitrate–nitrite–NO pathway as a distinct mechanistic axis linking oral ecology to vascular physiology. Particular emphasis is placed on factors influencing nitrate-reducing bacterial communities, interindividual variability in nitrate responsiveness, responder phenotypes, and emerging evidence regarding sex-related differences in nitrate metabolism and cardiovascular responses.

## 2. NO Generation Pathways and the Role of Oral Nitrate Metabolism

NO is generated in humans through two interconnected pathways whose relative contributions vary according to physiological context: the endogenous NOS pathway and the enterosalivary nitrate–nitrite pathway. Rather than functioning independently, these systems operate in a complementary manner, collectively regulating NO bioavailability across diverse oxygen, metabolic, and inflammatory conditions.

### 2.1. Endogenous L-Arginine/NOS Pathway

In mammals, the canonical source of NO is the L-arginine/NOS pathway, in which endothelial (eNOS), neuronal (nNOS), and inducible (iNOS) isoforms catalyze the oxygen-dependent conversion of L-arginine to NO and L-citrulline [1]. Among these, eNOS is the principal regulator of vascular homeostasis, contributing to endothelial integrity, vasodilation, and haemodynamic stability [22]. Efficient NOS activity requires adequate oxygen tension, sufficient intracellular L-arginine, and the availability of essential cofactors such as tetrahydrobiopterin (BH_4_) [22]. Under pathological conditions, including chronic inflammation, metabolic disorders, and oxidative stress, NOS function may become impaired [22,23]. Reduced cofactor availability and increased oxidative burden can promote eNOS uncoupling, shifting the enzyme from NO production toward superoxide generation, thereby decreasing NO availability and contributing to endothelial dysfunction [24,25]. In parallel, activation of iNOS during inflammatory responses leads to high-output NO production, which, in the presence of reactive species such as superoxide, may favor the formation of reactive nitrogen intermediates with potentially deleterious effects [26]. Collectively, these alterations highlight the NOS pathway’s sensitivity to pathological states and help explain why alternative mechanisms of NO generation become increasingly relevant in a wide range of chronic diseases.

### 2.2. The Enterosalivary Nitrate–Nitrite Pathway

**Figure 1:**
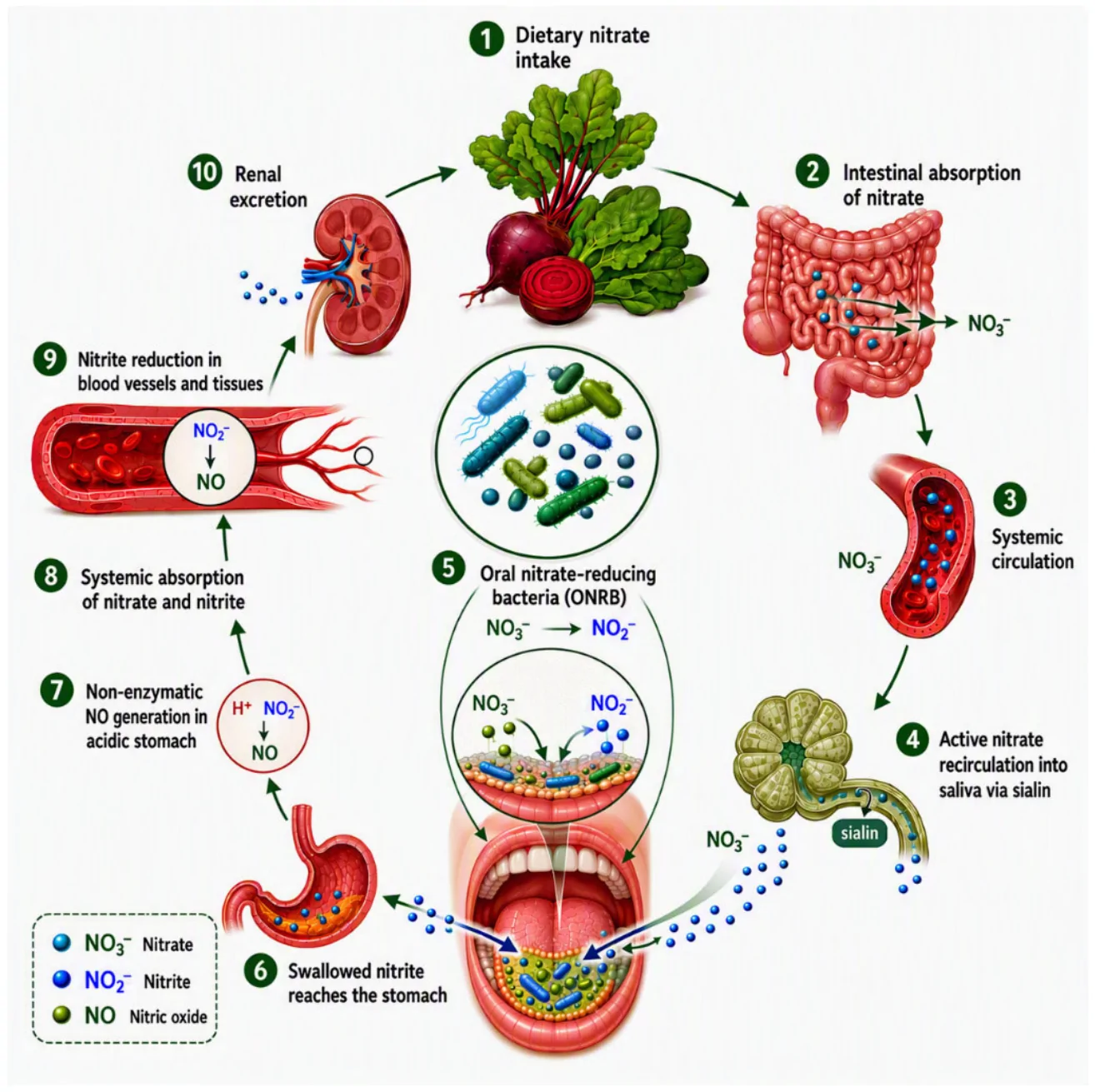
The enterosalivary nitrate–nitrite–NO pathway. Dietary nitrate is absorbed in the intestine and enters the systemic circulation. A portion of circulating nitrate is actively concentrated in saliva via the sialin transporter and subsequently reduced to nitrite by ONRB. Following swallowing, nitrite is converted to NO in the acidic gastric environment or absorbed into the circulation, where it serves as a substrate for NO generation in blood vessels and peripheral tissues. The remaining nitrate is cleared primarily through renal excretion.

In addition to enzymatic NO synthesis, humans rely on an alternative pathway that involves the sequential reduction in inorganic nitrate (NO_3_^−^), mainly from the diet to nitrite (NO_2_^−^) and subsequently to NO [27] (Figure 1). This enterosalivary pathway is critically dependent on the oral microbiome and represents a unique example of host–microbe cooperation in human physiology. Following intestinal absorption, nitrate is actively concentrated in saliva via the sialin (SLC17A5) transporter and reduced to nitrite by ONRB, including genera such as *Rothia*, *Neisseria*, *Actinomyces*, and *Veillonella* [28]. Because humans lack significant endogenous nitrate reductase activity, this microbial step constitutes a key determinant of nitrate bioactivation. The nitrite generated in the oral cavity can follow multiple pathways. A substantial fraction is swallowed and converted to NO in the acidic gastric environment through non-enzymatic reactions [29], while another fraction enters the systemic circulation and serves as a substrate for nitrite reduction in blood and tissues [3]. This latter process is mediated by several enzymatic systems, including deoxyhemoglobin, deoxymyoglobin, and xanthine oxidoreductase, and is particularly favored under conditions of reduced oxygen tension and altered tissue redox state [30]. Through these mechanisms, the enterosalivary pathway contributes to NO generation when oxygen-dependent NOS activity is limited [3,23]. Under hypoxic or acidic conditions, nitrite therefore serves as an important alternative source of NO, through enzymatic and non-enzymatic reduction pathways, thereby complementing the classical NOS-dependent pathway [3,27,30]. Nitrite reduction under these conditions contributes to the maintenance of NO homeostasis when oxygen availability is insufficient to support optimal NOS activity [3,27]. NO is a highly reactive and short-lived signaling molecule. Following its production, NO is rapidly oxidized to nitrite and subsequently to nitrate, which represent relatively stable circulating metabolites that can be recycled through the enterosalivary nitrate–nitrite–NO pathway [3,27]. This dynamic cycle highlights the physiological importance of nitrate and nitrite not merely as inert end products, but as bioactive reservoirs contributing to NO homeostasis [3,27]. Importantly, the efficiency of this pathway is influenced by the functional capacity of ONRB, linking oral microbial activity to systemic NO availability and highlighting nitrate-reducing capacity as a functionally relevant characteristic of the oral microbiome [31].

### 2.3. Determinants of Oral Nitrate Reduction

The nitrate-reducing capacity of oral bacteria is mediated by specialized nitrate reductase systems that enable the conversion of nitrate into nitrite, the key microbial step of the enterosalivary nitrate–nitrite–NO pathway. Among these, the membrane-bound respiratory nitrate reductase (Nar) and the periplasmic nitrate reductase (Nap) are the principal enzymatic systems involved in bacterial nitrate reduction. These enzymes differ in their cellular localization, regulatory mechanisms, and physiological functions, allowing bacteria to adapt nitrate metabolism to distinct environmental conditions [32,33].

**Figure 2:**
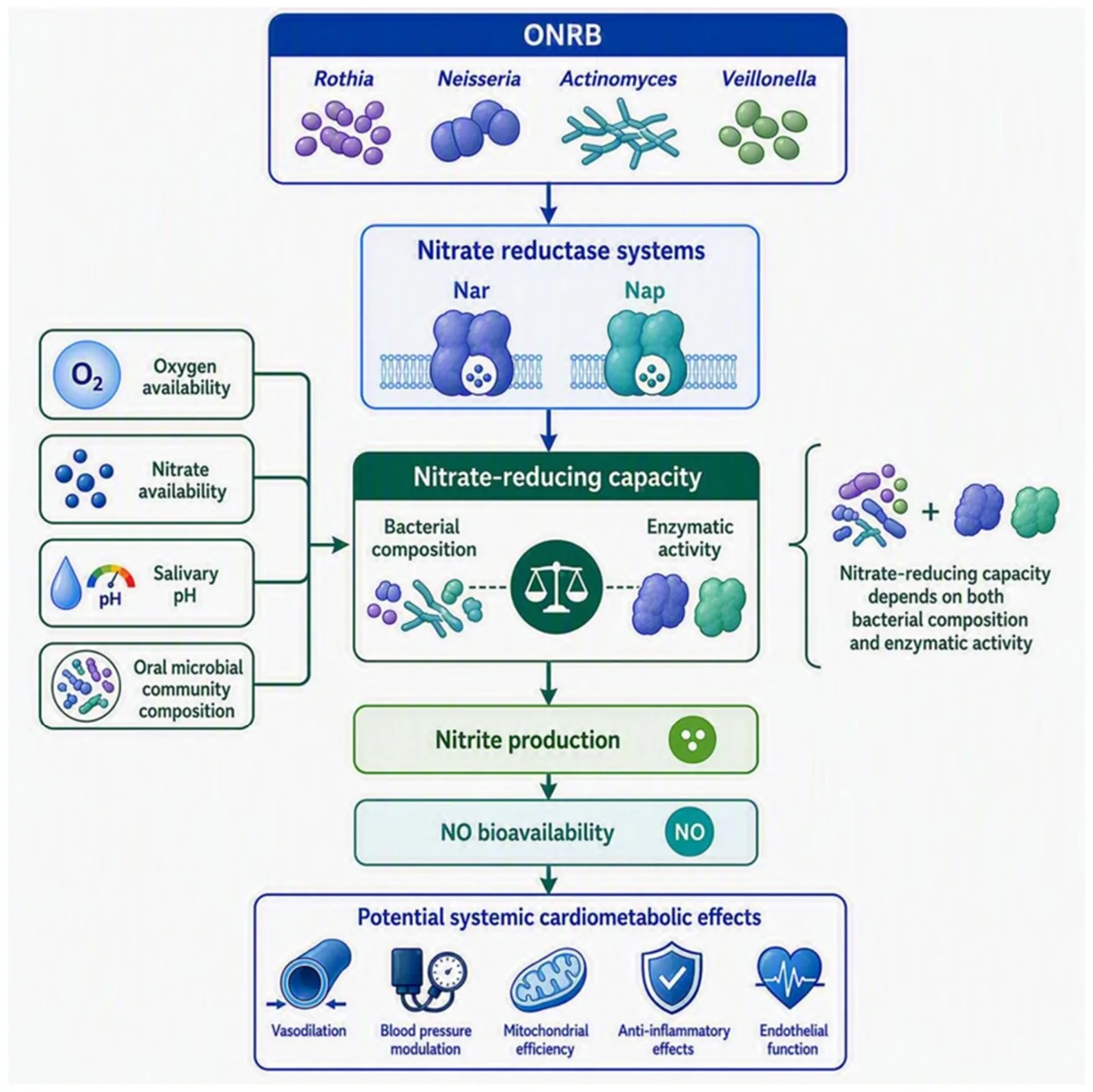
Determinants of ONRB. ONRB and their nitrate reductase systems (Nar/Nap) drive the conversion of nitrate to nitrite. This process is influenced by oxygen availability, nitrate availability, salivary pH, and oral microbial community composition, ultimately affecting NO bioavailability and potential cardiometabolic effects.

The distribution and expression of nitrate reductase genes vary among oral microbial communities, potentially contributing to interindividual differences in nitrate-reducing capacity and the subsequent generation of nitrite and NO metabolites. Furthermore, environmental factors such as oxygen availability, nitrate concentration, salivary pH, and microbial community composition can influence nitrate reductase activity and overall pathway efficiency [33,34] (Figure 2).

Increasing evidence suggests that the functional capacity of oral microbial communities may be as important as their taxonomic composition in determining nitrate bioactivation and subsequent NO generation. Consequently, a better understanding of the molecular mechanisms underlying oral nitrate reduction may support the development of microbiome-targeted strategies aimed at preserving or enhancing NO homeostasis and its associated cardiometabolic benefits [32,34].

### 2.4. Host–Microbe Cooperation in NO Homeostasis

The NOS-dependent and nitrate-dependent pathways form an integrated network that supports NO homeostasis. The relative contribution of each pathway is context-dependent: the NOS pathway predominates under normoxic conditions, whereas the nitrate–nitrite pathway becomes increasingly relevant under hypoxia, acidosis, or conditions associated with impaired NOS function [22]. Within this framework, the oral microbiome emerges as an important upstream regulator of the nitrate–nitrite pathway, providing a microbial contribution to NO homeostasis. Through the enterosalivary pathway, oral microbial activity complements endogenous NOS-dependent NO production, thereby extending the capacity of the host to maintain NO homeostasis under changing physiological conditions [35]. The coexistence of these pathways provides a degree of functional redundancy that may enhance the resilience of NO homeostasis across different physiological and pathological contexts.

Because NOS-dependent NO generation is highly sensitive to oxygen availability, enzymatic integrity, and cellular redox balance, endogenous NO synthesis may become compromised under conditions such as hypoxia, oxidative stress, chronic inflammation, and metabolic dysfunction [22,36]. Under these circumstances, the nitrate–nitrite pathway can continue to contribute to NO generation, providing an alternative source of NO that is less dependent on oxygen availability. Through this complementary organization, host and microbial pathways cooperate to preserve NO signaling despite environmental, metabolic, and disease-associated challenges. This integrated host–microbe perspective helps explain interindividual variability in responses to dietary nitrate and highlights the importance of oral ecological balance for maintaining efficient NO metabolism. Altogether, these pathways illustrate how oral microbial activity and host enzymatic systems cooperate to regulate NO availability across oral and systemic compartments.

In this sense, the oral microbiome contributes to a regulatory network that supports the stability and adaptability of NO homeostasis under changing physiological conditions.

## 3. Oral Dysbiosis, ONRB, and Systemic Implications

**Figure 3:**
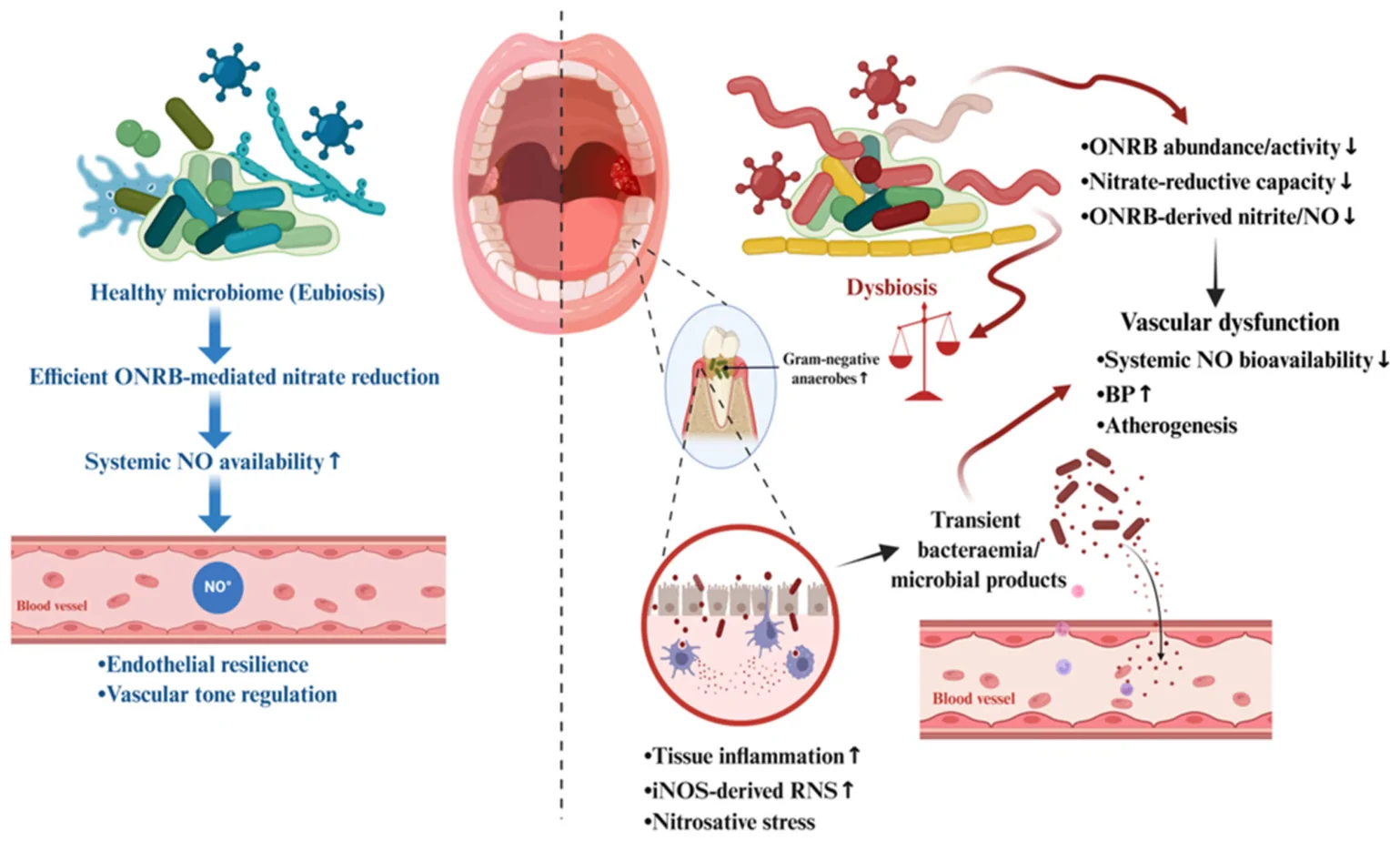
Oral microbiome–NO axis in eubiosis and dysbiosis. Left (Eubiosis): A healthy oral microbiome supports efficient nitrate reduction by ONRB, contributing to systemic NO formation via the enterosalivary pathway. This microbial contribution promotes endothelial resilience and vascular tone regulation. Right (Dysbiosis): Periodontal dysbiosis reduces ONRB abundance and nitrate-reducing capacity, leading to decreased nitrate-derived nitrite and NO availability. Dysbiotic communities enriched in Gram-negative anaerobes promote tissue inflammation, increase iNOS-derived RNS, and induce nitrosative stress. These alterations, together with the possible dissemination of microbial products, reduce systemic NO bioavailability and contribute to increased BP and early atherogenic changes. Upward arrows (↑) indicate an increase, whereas downward arrows (↓) indi-cate a decrease. Created in BioRender. Palumbo, P. (2026) https://BioRender.com/fxvqn7x.

The functional contribution of ONRB to nitrate-dependent NO homeostasis strongly influenced the ecological balance of the oral microbiome (Figure 3). Under eubiotic conditions, ONRB efficiently convert dietary nitrate into nitrite, thereby contributing to systemic NO generation through the enterosalivary pathway. This microbial contribution supports endothelial function, regulation of vascular tone, and overall NO homeostasis [1,2].

In contrast, oral dysbiosis disrupts this functional pathway at multiple levels. Reduced abundance and activity of ONRB impair nitrate reduction [37], leading to decreased nitrite availability and reduced NO bioactivation via the enterosalivary pathway [4,38,39]. This functional impairment occurs in parallel with proposed mechanisms linking periodontal disease to systemic conditions, including low-grade inflammation and transient dissemination of microbial products, rather than replacing them [40].

Importantly, dysbiotic communities enriched in periodontopathogenic Gram-negative anaerobes (e.g., *Porphyromonas gingivalis* and *Fusobacterium nucleatum*) promote a pro-inflammatory microenvironment within periodontal tissues [40]. This condition is associated with increased iNOS activity and the accumulation of reactive nitrogen species (RNS) [41], resulting in nitrosative stress and local tissue damage. In this context, NO signaling becomes dysregulated, shifting from a homeostatic mediator to a contributor to inflammatory and oxidative processes [4].

Collectively, these alterations suggest that impaired nitrate-reducing capacity is not merely a consequence of dysbiosis but may contribute to alterations in systemic NO homeostasis. Within this framework, disruption of microbial nitrate metabolism represents a potential mechanism linking oral dysbiosis to vascular dysfunction through reduced NO bioavailability and enhanced inflammatory and nitrosative responses [4,10,31,39].

### Periodontal Disease, Vascular Dysfunction, and Nitrate Metabolism

A growing body of evidence supports the association between periodontal disease and systemic conditions, particularly cardiovascular disorders [42]. Established mechanisms linking periodontal disease to vascular dysfunction include systemic inflammation, transient bacteremia, and immune activation [43]. Similar oral–systemic interactions have also been described in chronic kidney disease, where impaired oral health and periodontal alterations are increasingly recognized as contributors to systemic inflammatory burden and disease progression [44,45]. In addition to these pathways, impaired oral nitrate metabolism is emerging as a complementary, potentially underappreciated mechanism linking periodontal health to vascular regulation [43]. Reduced nitrate-reducing capacity in individuals with periodontal disease may contribute to decreased systemic NO availability, thereby influencing BP regulation, endothelial function, and vascular tone [39]. This concept is supported by clinical observations linking reduced abundance or activity of ONRB with altered hemodynamic responses, reduced NO bioavailability, and impaired BP regulation [5].

Importantly, this mechanism integrates microbial function with host vascular physiology, providing a functional bridge between oral dysbiosis and systemic outcomes. Although causality remains to be fully established, current evidence supports the view that impaired microbial nitrate reduction is not simply a bystander effect of periodontal disease but may represent a contributory pathway within the broader oral–systemic axis. In this perspective, nitrate-reducing capacity can be interpreted as a measurable functional trait of the oral microbiome with potential relevance for oral, vascular, and systemic health.

## 4. Behavioral and Environmental Modulators of ONRB

Beyond disease-associated dysbiosis, oral nitrate metabolism is dynamically regulated by a range of behavioral and environmental factors that influence the functional activity of ONRB. These modulators act by altering microbial composition, substrate availability, and enzymatic activity, thereby shaping nitrate reduction efficiency and systemic NO bioavailability.

Within this framework, both enhancing and disrupting factors can be identified. Dietary nitrate intake and microbiome-targeted strategies support ONRB activity and promote nitrate bioactivation, whereas antiseptic mouthwashes represent a well-characterized example of pathway disruption. Understanding the balance between these opposing influences contribute to interpreting interindividual variability in nitrate metabolism and its systemic effects.

### 4.1. Dietary Nitrate, ONRB, and BP Regulation

**Figure 4:**
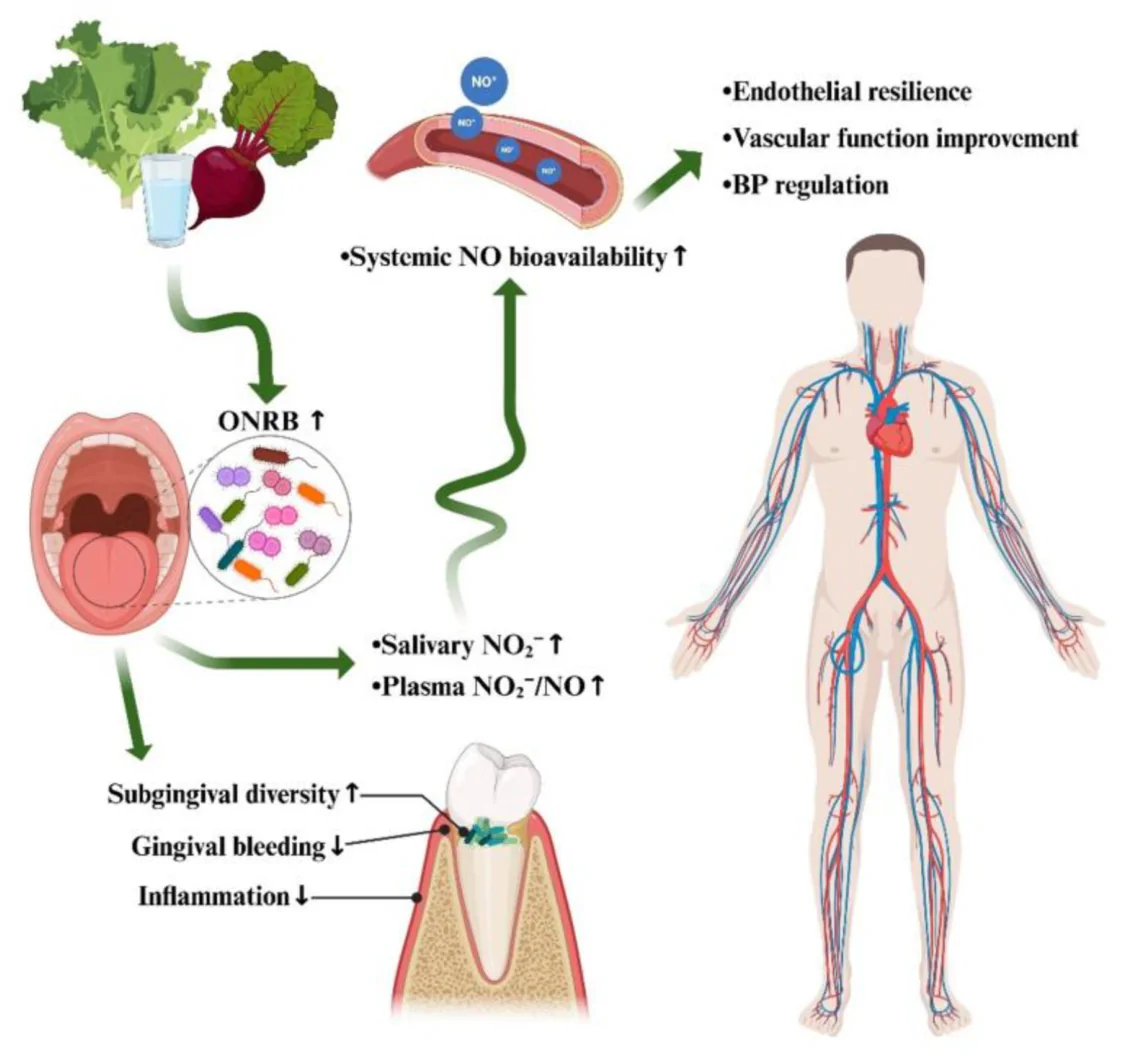
Effects of dietary nitrate on ONRB, nitrite-derived NO bioavailability, and vascular function. Dietary nitrate enhances the abundance and activity of ONRB, leading to increased salivary nitrite and plasma nitrite/NO levels. These microbial and biochemical changes promote subgingival microbial diversity, reduce gingival bleeding, and decrease local inflammatory burden. At the systemic level, enhanced nitrite-derived NO bioavailability supports endothelial function and contributes to BP regulation. Upward arrows (↑) indicate an increase, whereas downward arrows (↓) indi-cate a decrease. Created in BioRender. Palumbo, P. (2026) https://BioRender.com/fxvqn7x.

Dietary nitrate represents a non-pharmacological approach to support NO availability through the enterosalivary nitrate–nitrite–NO pathway, particularly in conditions where endogenous NOS-dependent NO production is reduced (Figure 4). Nitrate-rich foods, especially green leafy vegetables and beetroot, provide the substrate for microbial nitrate reduction and subsequent nitrite-dependent NO generation, while also influencing the composition and functional activity of the oral microbiome [9,26,38]. To provide an overview of the available human evidence, representative studies investigating oral nitrate-reducing bacteria, nitrate-based interventions, and nitric oxide-related oral and vascular outcomes are summarized in Table 1.

Growing clinical evidence indicates that dietary nitrate intake is associated with beneficial effects on oral health, mediated at least in part by nitrate-reducing bacteria. Randomized controlled trials have shown that nitrate-rich diets can reduce gingival inflammation and improve periodontal indices, even in the absence of major changes in plaque accumulation [46,47]. These findings suggest that microbial nitrate reduction and downstream NO generation may contribute to improvements in periodontal inflammatory status. Consistent with this interpretation, microbiome analyses show that nitrate intake enriches canonical nitrate-reducing genera, such as *Rothia* and *Neisseria*, increases subgingival microbial diversity, and promotes an oral ecological profile favorable to efficient nitrate reduction [38].

At the systemic level, dietary nitrate intake has been shown to shift the salivary microbiome toward nitrate-reducing profiles and to reduce BP, particularly in older adults and individuals with impaired vascular function [10]. Individuals with hypertension often exhibit reduced oral microbial diversity and lower abundance of ONRB [35,49], suggesting a diminished capacity for nitrate-to-nitrite conversion. Consequently, ONRB emerge as important modulators of the vascular response to dietary nitrate [50,51]. Additional trials further demonstrate that nitrate supplementation increases plasma nitrite levels and enriches nitrate-reducing taxa [16], whereas low-nitrate diets reduce salivary nitrate and nitrite concentrations and attenuate BP responses [12]. In high-risk cardiovascular populations, longer-term dietary nitrate intake has been associated with improvements in endothelial function, reductions in arterial stiffness, and enrichment of ONRB [48]. Together, these findings indicate that dietary nitrate modulates oral microbial function, systemic nitrite availability, and BP regulation, supporting both oral health and cardiovascular function.

### 4.2. Oral Microbiome-Dependent Variability in Vascular Responses

Despite consistent biochemical effects of dietary nitrate on circulating nitrate and nitrite levels, the physiological response, particularly in terms of BP reduction, shows substantial interindividual variability. Emerging evidence indicates that this variability is largely driven by differences in the abundance and functional activity of nitrate-reducing bacteria. Individuals with higher ONRB abundance or activity tend to exhibit greater increases in nitrite levels and more pronounced NO-mediated vascular responses following nitrate intake [11]. Conversely, individuals with oral dysbiosis or reduced nitrate-reducing activity may display attenuated physiological responses, even in the presence of adequate dietary nitrate [52]. This observation has led to the identification of microbial “responder” and “non-responder” phenotypes, in which oral microbiome function plays a central role in determining the effectiveness of dietary nitrate interventions [53]. These findings underscore the importance of considering oral microbial function when interpreting the systemic vascular effects of diet and help explain variability in clinical outcomes across intervention studies.

Although numerous studies support a beneficial role of dietary nitrate and ONRB-mediated nitrate bioactivation in cardiovascular health, some investigations have reported modest or non-significant vascular effects, and findings are not entirely consistent across investigations. Variability in participant characteristics, oral health status, nitrate dosage, intervention duration, and methods used to assess nitrate-reducing capacity may contribute to heterogeneous outcomes. Moreover, interindividual differences in microbial composition and functional activity may partly explain the presence of responder and non-responder phenotypes following nitrate supplementation [11,37,52,53]. Therefore, larger and more standardized clinical studies are needed to clarify the magnitude and clinical relevance of these effects.

### 4.3. Microbiome-Targeted Strategies to Enhance Nitrate Bioactivation

In addition to dietary nitrate intake, microbiome-targeted approaches are emerging as potential strategies to enhance nitrate bioactivation and downstream NO availability. These include the use of nitrate-reducing probiotic strains and symbiotic approaches that combine dietary nitrate with microbial supplementation. Experimental and early clinical studies suggest that such interventions can increase nitrate-to-nitrite conversion efficiency and amplify physiological responses associated with nitrite-dependent NO generation [14,15]. By supporting the functional capacity of ONRB, these approaches may enhance the effectiveness of dietary nitrate interventions.

Microbiome-targeted strategies may be particularly relevant for individuals with impaired oral microbial function, including those with periodontal disease, reduced microbial diversity, or frequent exposure to antiseptic agents. In these settings, restoration of nitrate-reducing capacity may help in re-establishing the link between dietary nitrate intake and systemic NO bioavailability. Although current evidence remains limited, and much of the available data derive from preclinical models or early-stage human studies, these findings highlight the potential of targeted oral microbiome modulation as a complementary approach to support nitrate metabolism and its associated vascular effects. Further adequately powered clinical trials are needed to establish the long-term efficacy, safety, and clinical applicability of these interventions.

### 4.4. Antiseptic Mouthwashes, Suppression of ONRB, and BP Effects

**Figure 5:**
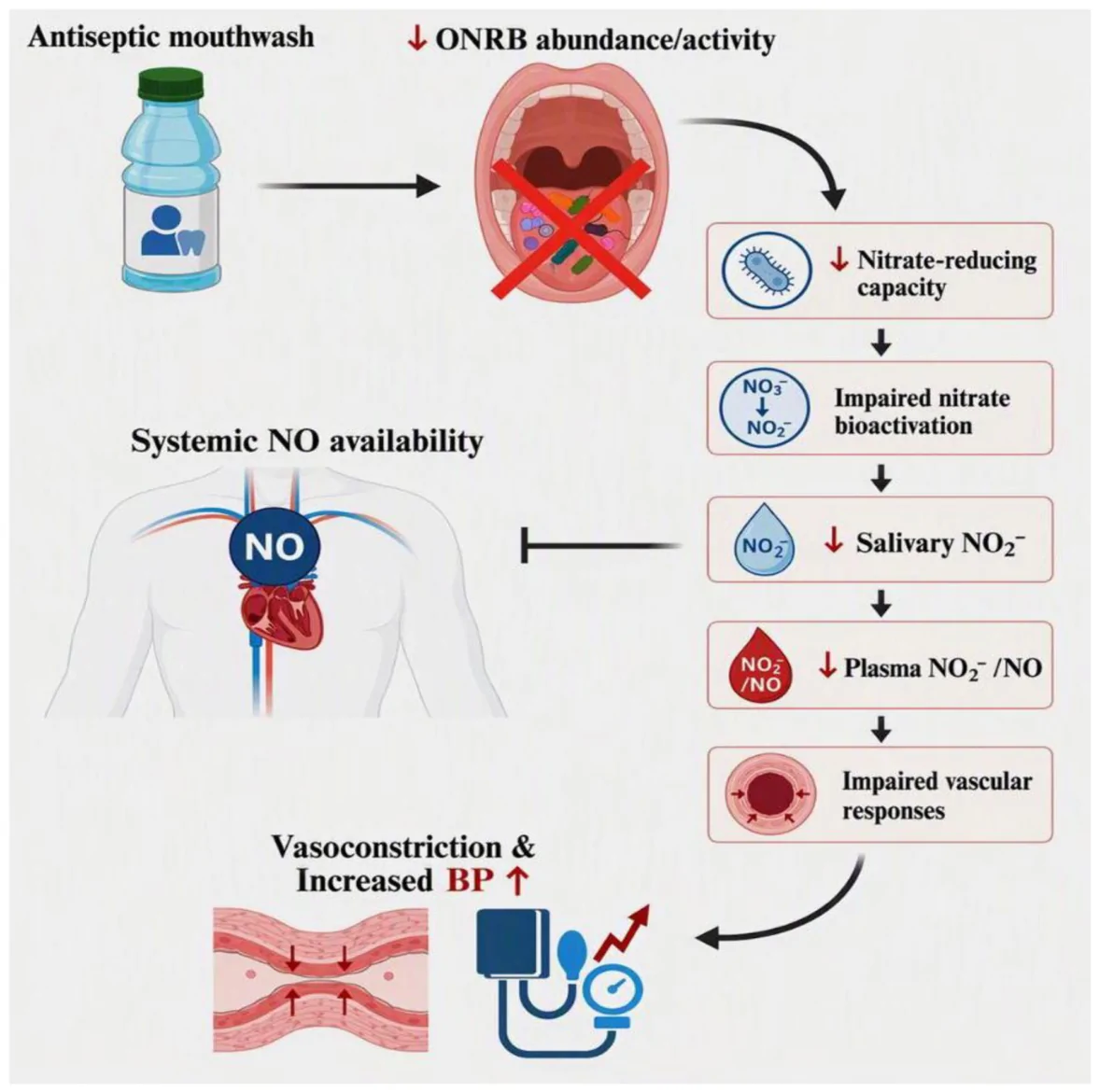
Antiseptic mouthwash-induced suppression of the ONRB–nitrite–NO pathway and its vascular effects. Antiseptic mouthwash reduces the abundance and activity of ONRB leading to impaired nitrate-reducing capacity and disruption of oral nitrate bioactivation. The resulting decreases in salivary nitrite and plasma nitrite/NO levels lower systemic NO availability, attenuate NO-mediated vascular responses, and promote vasoconstriction and increased BP. Upward ar-rows (↑) indicate an increase, whereas downward arrows (↓) indicate a decrease. Created in BioRen-der. Palumbo, P. (2026) https://BioRender.com/fxvqn7x.

The use of antiseptic mouthwashes provides a valuable interventional framework to investigate the functional role of ONRB in systemic NO regulation (Figure 5).

Short-term use of chlorhexidine-based formulations has been consistently shown to suppress ONRB abundance and activity, resulting in reduced salivary and plasma nitrite levels and measurable increases in BP [5,6]. These findings support the concept that the oral step of nitrate bioactivation represents an important step for systemic NO availability.

Suppression of ONRB is associated with attenuation of NO-dependent vascular responses, including blunted increases in nitrite following dietary nitrate intake and reduced vasodilatory responses [54]. Together, these observations provide evidence supporting that disruption of oral nitrate reduction can rapidly influence systemic vascular regulation. Observational studies further suggest that frequent use of antiseptic mouthwashes may be associated with a higher incidence of hypertension [55], although causality and long-term clinical relevance remain to be fully established. From a clinical perspective, these findings support a microbiome-aware approach to oral hygiene. While antiseptic mouthwashes remain appropriate for specific indications, such as postoperative care or acute infections, their routine and indiscriminate use may be undesirable, particularly in individuals with increased cardiometabolic risk [54,55].

Overall, evidence from antiseptic mouthwash interventions suggests that disruption of oral microbial function can extend beyond the oral cavity and influence systemic NO homeostasis. However, these effects should be interpreted within the broader context of physiological modulators of the pathway, particularly dietary nitrate availability, which plays a central role in sustaining microbiome-dependent NO bioactivation.

## 5. Sex as a Potential Modulator of Oral Nitrate Metabolism

Biological sex is a recognized determinant of immune regulation, microbial ecology, and vascular physiology, and emerging evidence suggests that these differences may also influence oral nitrate metabolism [56]. Sex-related differences have been extensively documented in both the gut microbiome and cardiometabolic responses to microbial metabolites [57,58,59], and recent evidence indicates that similar sex-based divergence also exists within the oral microbiome, with men and women showing distinct biofilm structures and microbial community profiles during periodontitis [60]. Despite this broader sexual dimorphism across host–microbe interactions, limited attention has been directed toward sex-specific differences in ONRB.

Available evidence suggests that sex may influence nitrate-reducing capacity and downstream NO bioavailability. Human studies report higher baseline nitrite levels and greater increases in nitrite following dietary nitrate intake in females, consistent with enhanced oral nitrate-reducing activity, even in the absence of marked differences in overall microbial composition [19,20]. In contrast, reduced abundance or activity of nitrate-reducing oral taxa has been observed in specific female populations with impaired BP regulation, suggesting that sex-related differences in oral microbial function may influence vascular responses [11,52].

Supporting the relevance of biological sex in oral–systemic interactions, Andrukhov et al. reported sex-related differences in nitric oxide production, systemic inflammatory markers, and lipid metabolism among patients with periodontitis [61]. These findings suggest that biological sex may modulate the relationship between oral inflammation and systemic responses, further supporting the concept that sex-specific factors can influence NO homeostasis and related cardiovascular outcomes.

Several biological factors may contribute to this variability. Hormonal modulation of oral microbial communities and mucosal immunity may shape ecological niches that support ONRB, while sex-specific inflammatory responses and differences in salivary flow or composition may further influence nitrate reduction efficiency [19]. In addition, baseline vascular sensitivity to NO may modulate the physiological impact of nitrate-derived NO generation.

Despite these plausible mechanisms, systematic data remain limited. Most studies involve small sample sizes, focus predominantly on microbial taxonomy rather than functional nitrate-reducing capacity, and are often restricted to female cohorts, leaving sex-comparative analyses incomplete. As a result, the contribution of biological sex to variability in the oral nitrate–nitrite–NO pathway remains insufficiently defined.

Future research incorporating sex-balanced study designs, functional assessment of oral nitrate-reducing capacity, and integration with vascular phenotyping will be essential to clarify the role of sex in oral nitrate metabolism. Improved understanding of sex-related variability may help explain interindividual differences in nitrate-reducing capacity and downstream vascular responses, supporting the development of more personalized microbiome-based approaches to oral and systemic health management.

## 6. Conclusions and Future Perspectives

The evidence reviewed herein supports a conceptual shift in how the oral microbiome is viewed within the oral–systemic axis. Beyond its taxonomic composition, the oral microbiome should be considered a functional metabolic system capable of influencing host physiology through nitrate reduction and subsequent NO generation. Within this framework, nitrate-reducing capacity emerges as an important functional characteristic of the oral microbiome, with potential relevance for vascular regulation and systemic health.

Current findings suggest that microbial nitrate metabolism may represent a complementary mechanistic pathway linking oral and systemic physiology, alongside established mechanisms involving inflammation and microbial dissemination. This perspective expands current understanding of host–microbe interactions by highlighting the contribution of oral microbial function to NO homeostasis and vascular biology.

Despite growing interest in this field, important questions remain unresolved. The determinants of interindividual variability in nitrate-reducing capacity are still poorly understood, and microbial composition alone may not adequately predict functional responses. Likewise, the influence of biological sex on oral microbial nitrate metabolism and downstream physiological outcomes remains insufficiently explored. These knowledge gaps emphasize the need to move beyond descriptive microbial profiling toward functional assessment of oral microbial activity.

**Figure 6:**
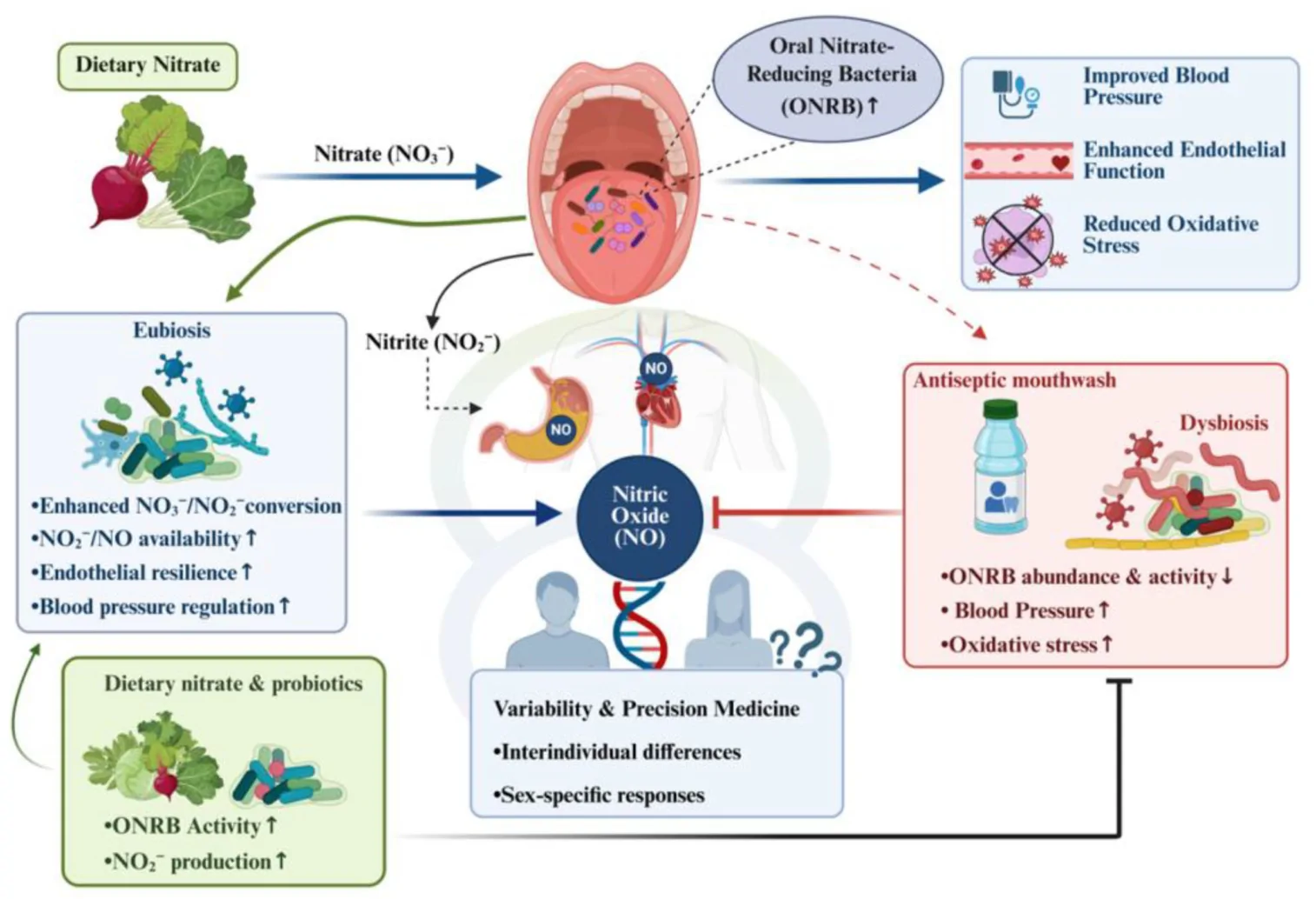
Schematic representation of the oral microbiome–NO axis, illustrating the role of ONRB in modulating NO bioavailability and vascular function. Dietary nitrate and microbiome-targeted strategies enhance ONRB activity and nitrate reduction, whereas antiseptic mouthwashes disrupt this pathway. Interindividual variability and sex-related differences may influence nitrate-reducing capacity and downstream physiological responses, supporting a precision medicine perspective. Upward arrows (↑) indicate an increase, whereas downward arrows (↓) indicate a decrease. Created in BioRender. Palumbo, P. (2026) https://BioRender.com/fxvqn7x.

Future research should prioritize functional measurements of nitrate-reducing capacity, integration of oral microbiome profiling with vascular phenotyping, and identification of responder and non-responder phenotypes. Such approaches may help clarify the biological significance of oral nitrate metabolism and support the development of personalized microbiome-targeted strategies aimed at optimizing NO bioavailability and systemic health. A schematic overview of the proposed framework and its principal modulators is presented in Figure 6.

Overall, recognizing oral nitrate metabolism as a functional interface between microbial ecology and host physiology provides a broader perspective on oral–systemic interactions and highlights new opportunities for integrating microbiome science into preventive and translational medicine.

## Figures and Tables

**Table 1:** Representative human studies investigating oral nitrate-reducing bacteria, nitrate-based interventions, and nitric oxide-related outcomes.

Reference	Population	Intervention	Main Microbiome Findings	Main Oral/Systemic Outcomes
[46]	Periodontal recall patients	Repeated nitrate-rich lettuce juice consumption	Enhanced nitrate–nitrite–NO metabolism	Reduced gingival inflammation
[47]	Subjects with gingivitis	Anti-inflammatory nitrate-rich dietary intervention	Not assessed	Improved gingival inflammatory parameters
[38]	Periodontal recall patients	Nitrate-rich diet	Increased abundance of nitrate-reducing taxa and microbial diversity	Ecological profile favorable to nitrate reduction
[10]	Healthy adults	Dietary nitrate supplementation	Shift toward a nitrate-responsive oral microbiome	Reduced BP; improved NO homeostasis
[16]	Healthy adults	Dietary nitrate supplementation	Enrichment of nitrate-reducing taxa	Increased plasma nitrite; enhanced vascular responsiveness
[12]	Healthy adults	Low- vs high-nitrate diet	Not assessed	Changes in salivary/plasma nitrate and nitrite levels; altered BP responsiveness
[48]	Hypercholesterolemic patients	Chronic dietary nitrate supplementation	Not assessed	Improved endothelial function and vascular health
[11]	Hypertensive women	Acute dietary nitrate supplementation	Altered nitrate-reducing bacterial profiles predicted responsiveness	Greater BP reduction in subjects with higher nitrate-reducing capacity

## Data Availability

No new data were created or analyzed in this study. Data sharing is not applicable to this article.

## Abbreviations

The following abbreviations are used in this manuscript:

NO	Nitric oxide
NOS	Nitric oxide synthase
ONRB	Oral nitrate-reducing bacteria
BP	Blood pressure
eNOS	Endothelial nitric oxide synthase
nNOS	Neuronal nitric oxide synthase
iNOS	Inducible nitric oxide synthase
Nar	Membrane-bound respiratory nitrate reductase
Nap	Periplasmic nitrate reductase
RNS	Reactive nitrogen species
